# Compression therapies for the treatment of venous leg ulcers: study protocol for a process evaluation in a randomised controlled trial, VenUS 6

**DOI:** 10.1186/s13063-023-07681-7

**Published:** 2023-11-14

**Authors:** Maartje Kletter, Jane Griffiths, Catherine Arundel, Jo Dumville, on behalf of the VenUS 6 Collaborators

**Affiliations:** 1 School of Health Sciences, The University of Manchester, Oxford Road, Manchester, M13 9PL UK; 2 York Trials Unit, Department of Health Sciences – Faculty of Science, University of York, York, YO10 5DD UK

**Keywords:** Compression therapies, Time to healing, Wound healing, Venous leg ulcer, Randomised controlled trial, Process evaluation

## Abstract

**Background:**

The VenUS 6 parallel-group randomised controlled trial (RCT) will compare the clinical and cost-effectiveness of compression wraps, two-layer compression bandage and evidence-based compression therapy, comprising of two-layers of hosiery or four-layer bandages, for healing time of venous leg ulcers. We will conduct an embedded process evaluation to evaluate the implementation of the trial and the various compression therapies and to gain a more in-depth understanding of trial participant and nursing staff views and experiences of these therapies.

**Methods:**

This process evaluation will be a mixed-method study, embedded into a wider RCT. Qualitative data will be collected through semi-structured individual in-depth interviews with trial participants and staff members. Quantitative data will be collected using patient questionnaires and case report forms that are part of the main trial data collection process. Interview transcripts will be analysed using the Framework Analysis and interview data will be integrated with quantitative RCT data using the RE-AIM framework and the Pillar Integration Process.

**Discussion:**

We describe the protocol for a process evaluation, designed to assess the implementation of the various venous leg ulcer compression therapies as evaluated in VenUS6, and the experiences of trial participants and nursing staff using these. This protocol provides one example of how an embedded mixed-method process evaluation can be conducted.

**Trial registration:**

ISRCTN 67321719 (https://doi.org/10.1186/ISRCTN67321719). Prospectively registered on 14 September 2020.

Recruitment Infographic SWAT—MRC Hub for Trials Methodology Research SWAT repository #116. Registered on 13 April 2020.

Retention Thank You Card SWAT—MRC Hub for Trials Methodology Research SWAT repository #119. Registered on 13 April 2020.

Retention Newsletter SWAT—MRC Hub for Trials Methodology Research SWAT repository #28. Registered on 01 July 2007.

Retention Pen SWAT—MRC Hub for Trials Methodology Research SWAT repository #92. Registered on 01 April 2019.

Protocol version: V1.5, 26 May 2022.

**Supplementary Information:**

The online version contains supplementary material available at 10.1186/s13063-023-07681-7.

## Background

Venous leg ulcers (VLU) are common, recurring open wounds on the lower leg, caused by impaired blood flow in diseased or damaged leg veins. Sluggish blood flow increases venous pressure, which can severely impair wound healing [1]. VLUs are a symptom of severe venous disease and are one of the most common complex wounds in the United Kingdom (UK) [2, 3].

Compression therapy is a guideline-recommended first-line treatment for VLUs, involving the application of tight bandages, stockings, or other systems around the leg [1]. The graduated pressure applied by these devices aims to improve blood flow up the leg veins, reduce venous hypertension and promote ulcer healing [4].

There is randomised controlled trial (RCT) evidence that compression therapy reduces time to healing compared with no compression in people with venous leg ulcers [5]. Further RCT evidence suggests that multi-layer compression therapy, using multiple bandages or stockings layered over each other on the leg, is more effective than those with a single layer [6–8]. Two-layer compression bandages have been developed to deliver the same full compression of four-layer bandages but with reduced bulk. More recently, adjustable hook-and-loop fastened compression wraps have been introduced. While compression stockings and compression wraps can be given to patients for self-application, bandages must be applied by trained staff [9]. However, as of yet, there is a lack of evidence for the effectiveness of these treatments.

**Fig. 1:**
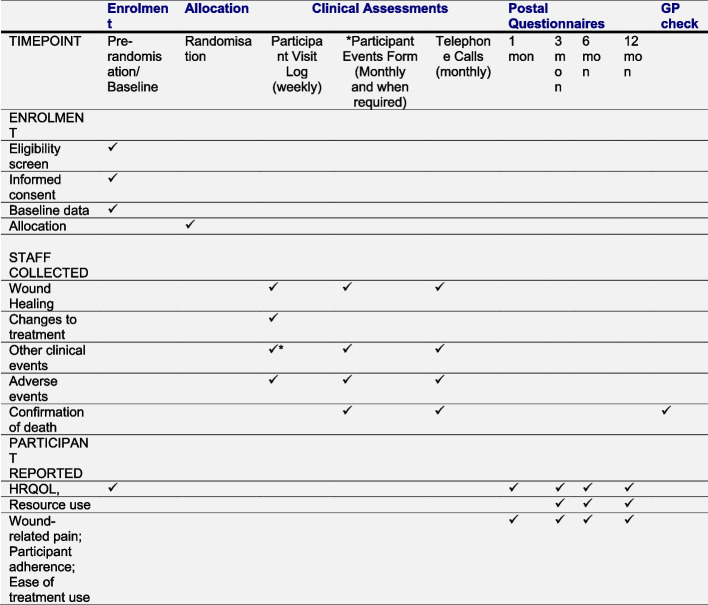
VenUS 6 SPIRIT figure

To generate further comparative evidence on the relative effects of compression therapies, the UK National Institute for Health and Care Research (NIHR) funded VenUS 6 [9, 10]. This three-arm parallel-group RCT will compare the clinical and cost-effectiveness of compression wraps and two-layer compression bandages with evidence-based compression, consisting of either four-layer bandages or two-layer compression stockings [9]. Figure 1 provides an overview of the data that will be collected as part of the main RCT. The primary outcome measure will be time to healing of the reference ulcer, while secondary outcomes include, amongst others, changes to allocated treatment and reasons for changes, health-related quality of life, adherence to treatment and ulcer-related pain [11].

Compression therapy can be considered a complex intervention, involving both the devices themselves and the requirements of staff and patients, regarding application of the therapy. The UK Medical Research Council (MRC) recommends that relative effectiveness estimates from RCTs of complex interventions should be interpreted alongside process evaluation findings [12]. A process evaluation aims to better understand the reality of intervention implementation and further explore causal mechanisms and contextual factors that help shape RCT outcomes [12]. The process evaluation can help explain discrepancies between hypotheses and observed outcomes in the RCT, may shed light on the ‘black box’ of complex interventions, provide information to interpret outcome results and aid future implementation of research findings into practice. A process evaluation can thus help distinguish between interventions that are inherently faulty and those that are badly delivered [13]. Combining process evaluations with RCTs can enable the development of detailed understandings of causality that can support a policymaker or practitioner in interpreting effectiveness data [12]. Apart from explaining trial outcomes, a process evaluation can also shed light on trial processes, allowing researchers to intervene during data collection. For example, in a 2007 RCT regarding a computerised decision support tool for patients with atrial fibrillation, one of the trial arms was discontinued, after the process evaluation showed patients did not understand the exercise offered as part of the discontinued arm [14].

In this study, we aim to evaluate the implementation of the various compression therapies for the treatment of venous leg ulcers to gain a more in-depth understanding of trial participant and nursing staff views and experiences of various compression therapies, and to help consider how these views and experiences may explain VenUS6 outcomes. Additionally, we aim to learn more about the experiences of staff and participants regarding trial implementation. The process evaluation will be informed by the RE-AIM implementation framework [15].

## Methods

This process evaluation is a mixed-method study nested within VenUS 6 [11]. Qualitative data will be collected through semi-structured individual in-depth interviews with trial participants and nursing staff at trial sites. Quantitative data will be collected using patient questionnaires and case report forms that are part of the main RCT data collection process, as explained in Fig. 1 [11]. As quantitative data collection and analyses are detailed in the trial protocol [9–11]; this paper focuses on qualitative data collection, analysis and synthesis of qualitative and quantitative data. We used SPIRIT reporting guidelines for this manuscript, and a SPIRIT checklist is provided in supplementary materials (Additional file 1) [16]. Quantitative and qualitative findings will be integrated with the help of the Pillar Integration Process. Additionally, the RE-AIM framework [15] will be used to guide the synthesis of findings regarding the implementation of the trial and compression therapy.

### Trial participant sampling and recruitment

We will contact VenUS6 participants who, at trial recruitment, consented to be approached about participation in interviews. Details of participants who have provided consent along with their contact details and information about the group they were randomised to will be transferred securely via encrypted email from York Trials Unit (YTU) to MK at The University of Manchester. A purposive sampling process will be used with the aim of including a sample with maximum variation regarding age, gender and pain experienced. We aim to conduct interviews until data saturation has been reached. As a previous study explored people’s experiences of, largely, compression bandages and hosiery use [17], we may consider oversampling of participants who have been randomised to compression wraps within this study, as less evidence is available regarding implementation of and experience with compression wraps.

Data will be reviewed regularly to determine if sufficient data has been collected to answer the research question. If there are gaps or topics that require additional exploration further interviews may be conducted accordingly.

### Nursing staff sampling and recruitment

We will aim to include a variety of staff members involved in trial recruitment and providing compression treatment at VenUS6 sites. In the interviews with staff, we will aim to learn more about the context of providing compression treatment and the implementation of trial processes at participating sites, including recruitment, trial participants switching treatment after randomisation and trial participant withdrawal. We will recruit until we believe we have reached data saturation and are no longer identifying new themes.

Staff will be approached by their site principal investigator who will provide them with a brief description of the qualitative study and the participant information leaflet. If the nurses are willing to participate their contact details and consent will be collected by YTU and provided via secure encrypted email to The University of Manchester. Subsequently, potential participants will be called to arrange a date and time for the interview. Additionally, we will invite some site Principal Investigators and nurses based in the site study teams to participate in an interview, to learn more about trial processes at various sites.

In addition to the interviews, we will conduct a service evaluation with the help of an anonymous survey (Additional file 3), aimed to obtain wider information about the following: how easy staff find it to apply the various compression treatments that are part of VenUS6, how staff access compression therapies and how long it takes for the treatment to arrive. Finally, we will ask them how easy they think patients normally find applying compression wraps or two-layer hosiery themselves.

### Data collection

Interviews will be primarily held via telephone, but videoconference interviews will also be offered if participants prefer this and have access to the appropriate technology.

With permission of participants, the semi-structured individual in-depth interviews will be recorded with an encrypted recording device. As participants will have provided written informed consent to be contacted for an interview previously, participants will be asked to reconfirm consent before the start of the interview. This will be documented in a separate recording, which will be saved in an encrypted file in a password-protected trusted and secure Research Storage Area at the University of Manchester. Interviews will be transcribed by one of the researchers.

Full topic guides are available in supplementary materials (Additional file 2). During trial participant interviews, we will aim to learn more about participants’ experiences with their leg ulcer, experiences with their assigned compression therapy and their views on trial participation. The following topics will be discussed:

- Experiences of leg ulcer(s)
- Experiences of study enrolment and involvement
- Experiences with compression therapy
- Reasons for non-adherence (if applicable)

Interviews with staff members will be aimed at learning more about staff experiences with providing compression therapy, including training provided. Additionally, we will discuss patient adherence to therapy. We will ask participating nurses about their experiences of trial recruitment and trial involvement as well as contextual factors that potentially impacted trial participation, adherence and compression therapy provided. The following topics will be discussed:

- Perceptions and experience of compression therapy
- Adherence to compression therapy
- Education and training provided to staff members
- Experiences of trial recruitment
- Experiences of trial involvement

The survey will be developed in Qualtrics and distributed via YTU networks.

### Data analysis

We will complete the process evaluation data analysis before the trial outcomes are known, as recommended by Moore et al. to avoid biased interpretation. Qualitative data will be analysed with the help of the Framework Method for analysis of qualitative data, as developed by Ritchie and Spencer [18] and applied to healthcare research by Gale et al. [19]. The method developed by Ritchie consists of five key stages: familiarisation, identification of thematic framework, indexing, charting and mapping and interpretation [18]. Gale et al. added two stages to better coordinate the process, including transcription and coding [19]. The framework method consists of clear steps to be followed during data analysis, which leads to highly structured outputs of summarised data, providing a starting point for the integration of qualitative and quantitative data [19].

An initial coding framework will be developed after the first four interviews have been conducted. Interview findings will be discussed with the Trial Management Group and where required actions will be undertaken with individual sites or across the trial as needed.

Staff and patient interview outcomes will be analysed according to the same analytical framework. The seven stages of the framework method are summarised in Table 1 [19].

**Table 1:** Overview of framework method as applied to the process evaluation in VenUS6

Stage	Description
Transcription	Audio recordings will be transcribed verbatim. To provide a contextual background of the interview process for telephone interviews notes regarding silences and interruptions will be taken
Familiarisation	Interview transcripts will be read and re-read, and notes regarding deviant cases or contrasting views of participants, including very strong opinions, will be taken. A short memo will be written about each interview transcript
Coding	Initially, four transcripts will be coded in NVivo. Two transcripts will be randomly selected to be independently coded by a second coder, to check for consistency. Coding will be discussed among the researchers and any discrepancies will be resolved
Developing framework	Codes for the first four transcripts will be integrated into categories, based on similarity, to develop an analytical framework. The developed framework will first be applied to the coded transcripts to assess fit or lack of fit. The fit will be discussed, and the analytical framework will be adapted based on the outcomes of the discussion. The developed framework will subsequently be presented to the Patient and Public Involvement (PPI) members and the Trial Management Group (TMG) and adapted according to discussion outcomes
Applying framework	The adapted analytical framework will be applied to index transcripts using the codes and categories as defined
Charting	Once all transcripts have been coded, the various codes will be charted into a matrix in Microsoft Excel. Each category will be listed as a column, with interview participants listed in the rows. Data from transcripts will be added to the matrix. The developed matrix will be discussed among the researchers and adapted according to the outcome of these discussions
Interpretation	Categories will be integrated into themes, based on similarity. Per theme, a memo will be developed, including a summary of data relevant to the theme, potential convergence and divergence among participants, as well as the categories and codes involved in the theme. The memo will contain some further points for consideration and potential gaps, and developed memos will be discussed among the researchers before they are presented to the TMG and PPI members for their comments. Memos will be adapted according to the outcomes of the discussions

The survey will be analysed with the help of descriptive statistics.

### Data synthesis

#### Pillar Integration Process

The Pillar Integration Process (PIP) [20] will be used to integrate the quantitative and qualitative findings. PIP consists of four stages, as outlined in Table 2. We will use the RE-AIM framework to guide data synthesis for findings regarding the implementation of the various compression therapies as well as trial implementation [15].

**Table 2:** Pillar Integration Process stages as applied to the process evaluation in VenUS6 [20]

Stage	Description
Listing	Five columns will be developed in Microsoft Excel, two columns on the left for quantitative findings and quantitative categories (grouped data), a central column for the ‘pillars’ and two columns on the right for qualitative findings, including a column for themes and a column including quotes that illustrate the themes. In collaboration with the trial statistician, hypothesised quantitative data will be listed and, if possible, categorised based on similarity
Matching	Qualitative data will be matched to the listed quantitative data. Qualitative data, including quotes, will be added in the opposite qualitative column. In each row, the qualitative items will reflect patterns, parallels, similarities or differences with the quantitative findings. Quantitative and qualitative findings that do not have a match will be left blank, highlighting gaps in the data
Checking	Once all data are matched, the developed matrix will be checked for consistency and to ensure no appropriate match could be provided for unmatched quantitative and qualitative findings. The lists and matches will be refined and/or modified based on the discussion
Pillar Building	Findings developed in the previous stages will be compared to develop the pillars. Memos, describing inferences about patterns, insights and themes that have emerged will be developed and summarised into the ‘PILLAR’ column, which will contain the integrated findings from each row. Pillars will be discussed with researchers before they are presented to the TMG and PPI members for their input. Pillars will be finalised according to the outcomes of the discussions

#### Logic model development

**Fig. 2:**
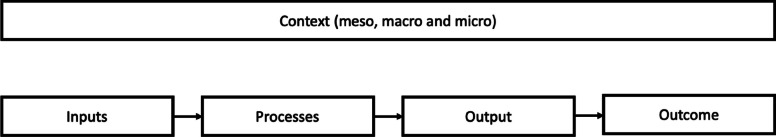
Overview of logic model, based on Petersen et al. and Ebenso et al. [21, 22]

Findings will be presented in a logic model as per Petersen et al. and Ebenso et al., shown in Fig. 2 [21, 22]. The logic model will contain important inputs, including staff and patient inputs, that feed into processes. These processes will subsequently lead to, or prevent, intermediate outcomes, also called outputs, and ultimate outcomes. All of this is influenced by contextual factors, which act at meso, macro and micro levels.

## Discussion

We described the protocol for a process evaluation, designed to assess the implementation of the various compression therapies evaluated in VenUS 6, and experiences of trial participants and nursing staff with the various compression therapies. In this protocol, we described how the qualitative data will be collected and integrated with quantitative data using the Pillar Integration Process.

A strength of the presented process evaluation is the embedded mixed method approach, where qualitative data are collected within a wider, well-resourced RCT complemented with qualitative data. The qualitative data will help tell the story behind the quantitative data, explaining the trial processes and outcomes and potentially providing some insight into what helped shape RCT outcomes. An additional strength is our use of RE-AIM [15] to guide data synthesis and the PIP, which will provide a structured way of integrating qualitative and quantitative findings, for integration of qualitative process evaluation findings with quantitative RCT findings. Additionally, the Framework Analysis will support the integration of data in the data synthesis stage.

The process evaluation will allow us to identify reasons for the lack of recruitment or, for example, for large numbers of non-adherence to assigned compression treatments. The process evaluation will start when the recruitment of participants in the wider trial is still taking place, allowing us to intervene at some sites where recruitment may be lacking. A further strength of the study will be MK’s outsider status for the day-to-day running of the trial and treatment for participants. MK will not be involved in trial recruitment, assigning treatment or providing treatment. We hope this will encourage participants to share their experiences with MK, which is a strength of the study.

One limitation includes selection bias, as we will only be able to include those trial participants who have consented to be approached for participation; indeed, the number of each type of participant we intend to recruit precludes us from recruiting from every participating site. Furthermore, those who have consented may be more enthusiastic about trial participation than those who did not consent.

In conclusion, this process evaluation protocol provides one example of how an embedded mixed-method process evaluation can be conducted. We hope that the process evaluation will provide us with a more in-depth understanding of the implementation process of various compression therapies and trial participant and staff experiences with the compression therapies, as well as insight into potential issues relating to trial processes, such as recruitment, adherence and withdrawal.

## Trial status

The trial is ongoing. Recruitment for the main trial commenced on 03 February 2021. The expected recruitment completion date is April 2024.

### Supplementary Information

**Additional file 1.** SPIRIT checklist.

**Additional file 2.** Venus 6 Process Evaluation Topic Guides.

**Additional file 3. **Anonymous survey. 

## Data Availability

Datasets and statistical code used in this study will be available from the corresponding author upon reasonable request following completion of the trial.

## Abbreviations

MRC: Medical Research Council
NIHR: National Institute for Health Research
PPI: Patient and Public Involvement
RCT: Randomised controlled trial
TMG: Trial Management Group
UK: United Kingdom
VLU: Venous leg ulcer
YTU: York Trials Unit

## References

[1] Excellence NIfHaC. Leg ulcer - Venous. 2021. Available from: https://cks.nice.org.uk/topics/leg-ulcer-venous/. Accessed 11 May 2023.

[2] Hall J, Buckley HL, Lamb KA, Stubbs N, Saramago P, Dumville JC (2014). Point prevalence of complex wounds in a defined United Kingdom population. Wound Repair Regen.

[3] Gray TA, Rhodes S, Atkinson RA, Rothwell K, Wilson P, Dumville JC (2018). Opportunities for better value wound care: a multiservice, cross-sectional survey of complex wounds and their care in a UK community population. BMJ Open.

[4] Woo KY, Alavi A, Evans R, Despatis M, Allen J (2013). New advances in compression therapy for venous leg ulcers. Surg Technol Int.

[5] Shi C, Dumville JC, Cullum N, Connaughton E, Norman G. Compression bandages or stockings versus no compression for treating venous leg ulcers. Cochrane Database Syst Rev. 2021;7:CD013397. 10.1002/14651858.CD013397.pub2 PMC8407020 34308565

[6] O'Meara S, Cullum N, Nelson EA, Dumville JC. Compression for venous leg ulcers. Cochrane Database Syst Rev. 2012;11:CD000265. 10.1002/14651858.CD000265.pub3 PMC7068175 23152202

[7] O'Meara S, Tierney J, Cullum N, Bland JM, Franks PJ, Mole T (2009). Four layer bandage compared with short stretch bandage for venous leg ulcers: systematic review and meta-analysis of randomised controlled trials with data from individual patients. BMJ.

[8] Ashby RL, Gabe R, Ali S, Saramago P, Chuang LH, Adderley U, et al. VenUS IV (Venous leg Ulcer Study IV) - compression hosiery compared with compression bandaging in the treatment of venous leg ulcers: a randomised controlled trial, mixed-treatment comparison and decision-analytic model. Health Technol Assess. 2014;18(57):1–293, v-vi. 10.3310/hta18570 PMC4781202 25242076

[9] Dumville JC. VenUS 6: A randomised controlled trial of compression therapies for the treatment of venous leg ulcers. Stud Protoc. 2021.10.1186/s13063-023-07681-737964358

[10] Dumville JC, Griffiths J, Watson J, Soares MO, Saramago P, Atkinson RA, et al. A randomised controlled trial of compression therapies for the treatment of venous leg ulcers. National Institute for Health Research. 2022. Available from: https://fundingawards.nihr.ac.uk/award/NIHR128625. Accessed: 11 May 2023.

[11] Arundel C, Welch C, Saramago P, Adderley U, Atkinson RA, Chetter I, et al. A randomsied controlled trial of compression therapies for the treatment of venous leg ulcers (VenUS 6): study protocol for a pragmatic, multicentre, parallel group, three arm randomised controlled trial. Trials. 2023;24:357. 10.1186/s13063-023-07349-2 PMC10223923 37237393

[12] Moore GF, Audrey S, Barker M, Bond L, Bonell C, Hardeman W (2015). Process evaluation of complex interventions: Medical Research Council guidance. BMJ.

[13] Oakley A, Strange V, Bonell C, Allen E, Stephenson J, Team RS (2006). Process evaluation in randomised controlled trials of complex interventions. BMJ.

[14] Murtagh MJ, Thomson RG, May CR, Rapley T, Heaven BR, Graham RH (2007). Qualitative methods in a randomised controlled trial: the role of an integrated qualitative process evaluation in providing evidence to discontinue the intervention in one arm of a trial of a decision support tool. Qual Saf Health Care.

[15] Glasgow RE, Harden SM, Gaglio B, Rabin B, Smith ML, Porter GC (2019). RE-AIM planning and evaluation framework: adapting to new science and practice with a 20-year review. Front Public Health.

[16] Chan AW, Tetzlaff JM, Gotzsche PC, Altman DG, Mann H, Berlin JA (2013). SPIRIT 2013 explanation and elaboration: guidance for protocols of clinical trials. BMJ.

[17] Perry C, Atkinson RA, Griffiths J, Wilson PM, Lavallee JF, Cullum N, et al. Barriers and facilitators to use of compression therapy by people with venous leg ulcers: a qualitative exploration. J Adv Nurs. 2023;79(7):2568–84. 10.1111/jan.15608 36811300

[18] Ritchie J, Spencer L. Qualitative data analysis for applied policy research. In: Bryman A, Burgess R, editors. Analyzing qualitative data. London: Routledge; 1994.

[19] Gale N, Heath G, Cameron E, Rashid S, Redwood S. Using the framework method for the analysis of qualitative data in multi-disciplinary health research. BMC Med Res Methodol. 2013;13:117. 10.1186/1471-2288-13-117 PMC3848812 24047204

[20] Johnson R, Grove A, Clarke A (2017). Pillar Integration Process: a joint display technique to integrate data in mixed methods research. J Mixed Methods Res.

[21] Petersen D, Taylor E, Peikes D. The logic model: the foundation to implement, study, and refine patient-centered medical home models. Rockville: Agency for Healthcare Research and Quality 2013.

[22] Ebenso B, Manzano A, Uzochukwu B, Etiaba E, Huss R, Ensor T (2019). Dealing with context in logic model development: reflections from a realist evaluation of a community health worker programme in Nigeria. Eval Program Plann.

