# Supplementary material for: Compression therapies for the treatment of venous leg ulcers: study protocol for a process evaluation in a randomised controlled trial, VenUS 6
Source: Trials. 2023 Nov 14;24:727. doi: 10.1186/s13063-023-07681-7 (PMC10644437; doi:10.1186/s13063-023-07681-7)
Supplement: Supplementary file 2 — Additional file 2. Venus 6 Process Evaluation Topic Guides. [file 13063_2023_7681_MOESM2_ESM.pdf]

This topic guide covers three overarching areas that will be discussed in a conversational style interview: experience of the ulcer, involvement in the study, and experience of the compression treatment. Example questions to draw out the participant and suggested prompts are included. The interviews are iterative however and will be responsive to other issues raised by the participant where relevant to the aims and objectives of the study.

Venus 6 interview topic guide: Participants

Introduction

- We have approached you to share your experiences of being involved in the Venus 6 leg ulcer study.
- The purpose of the interview is to give you the opportunity to talk about your experiences of being involved in the study, and the compression therapy you have been given for your leg/s.
    - PROMPT: Can you tell me more about….. ; can you give an example?

Topics

1. Experience of Leg Ulcer

- I wondered if I could start by asking you a little about your leg ulcer?
    - PROMPT: When did it start? How have you been taken care of? By whom and using which compression therapy? Have you had any other problems at all related to your condition?
    - PROMPT: Have you tried anything yourself to manage your condition? Has this been successful, if so how? If this hasn't been successful, how not?

2. Enrolment/Involvement

- I am interested in how you were asked about taking part in the study?
    - PROMPT: What were your thoughts on the information you received? Was there any other information you would have liked to receive when deciding to participate?
    - PROMPT: What were your thoughts when you heard about the research?
- Why did you choose to participate in the research study?
- How was your compression therapy explained to you? By who?
    - PROMPT: Did you understand what was said?
- Did they explain how you would be allocated to your compression therapy?
    - PROMPT: Do you remember what was said?

3. Experience of Compression

- I am interested in your experience of your compression therapy
- Which compression do you currently have on your leg/s?
- What was your initial compression therapy preference? /What group did you want to be allocated to? Why was this your preference?
- Has you therapy preference changed at all?

- Do you wear them day and night?
- Who applies your compression?
  - PROMPT: If the patient or carer, how easy is this? How are you finding applying your compression therapy?
- What is your experience of the compression therapy?
  - PROMPT: How are you finding it? How does it affect your daily life? Does it affect your ability to care for yourself? Reduce your mobility? Ability to get out and about?
  - PROMPT: How does this compression therapy compare with previous treatments? Is it more or less painful or uncomfortable? Does it stay in place more or less than your previous compression? Do you notice a difference between the day and night using this compression therapy?
  - PROMPT: Are there any other benefits to it? Do you have any other difficulties with it?

FOR PARTICIPANTS USING COMPRESSION WRAPS ONLY:

- How are you finding managing your own care?
  - PROMPT: What is going well? Less well?
  - PROMPT: How have the nurses supported you with self-management? How have you/carers overcome any problems you/carers are having with your compression?
  - PROMPT: Have you ever removed some or all of your bandages/wrap? Why did you/carer remove some or all of your bandages/wrap?
- What motivates/helps you to continue with the compression therapy?
  - PROMPT: Have you ever thought about stopping compression therapy?

FOR NON-ADHERENT/ THOSE WHO HAVE CHANGED TREATMENT ONLY:

- I understand you have stopped/changed your compression therapy. Why is this?
  - PROMPT: Was it painful or uncomfortable? Did you feel that it did not stay in place as well as previous compression? Were you experiencing difficulties with self application (if relevant)? Did you prefer to have a different compression therapy?

This topic guide covers four overarching areas that will be discussed in a conversational style interview: involvement in the study, perceptions and experiences of compression, adherence and education and training. Example questions to draw out the participant and suggested prompts are included. The interviews are iterative however and will be responsive to other issues raised by the participant where relevant to the aims and objectives of the study.

Venus 6 Interview topic guide: staff

Introduction

- We have approached you to share your experiences of being involved in the Venus 6 leg ulcer study.
- The purpose of the interview is to give you the opportunity to talk about your experiences of being involved in the study and of your patients' experiences of the compression therapies.

Topics

1. Involvement in the study

- Can you talk to me about how you became involved in the study?
- How have you found your involvement so far?
  - PROMPT: Any challenges? What has worked well/less well?
- What has been your experience of treating study participants? How have you found the application of the different compression therapies?

2. Perceptions and experiences of compression therapy

- What is your perception of patients' experiences of the compression therapies? Are any of them better tolerated than others? Why do you think this is?
- For those patients using compression wraps - what is your perception of how they are managing their own care? Carers support?
  - PROMPT: What is going well? Less well?
  - PROMPT: How are you supporting them with self-management?
- How do you think patients are experiencing the different compression therapies? How does the compression therapy affect daily life? Do they affect ability to self-care? Reduce their mobility? Ability to get out and about?
- How do you think patients are experiencing the different compression therapies? How does the compression therapy affect daily life? Do they affect ability to self-care? Reduce their mobility? Ability to get out and about?
- How do the allocated compression therapies compare to previous compression/treatment for your patients? e.g. do they appear to be more or less painful or uncomfortable? Do they stay in place more or less than other compression therapies?
- How do you support patients with any problems they are having with their compression therapy?
- What do you think motivates/helps patients to continue with the compression therapy?
- What do you think motivates healthcare professional to provide certain compression therapies for their patients?

- For patients that have crossed over to a different compression therapy, why do you think this is?
- How well do you think patients understand the underlying pathology that leads to the ulcer?
- Do they receive adequate education on/explanation about this? What are they taught, by whom and how? What do you think would make this clearer for patients?

3. Adherence to Compression

- What is your perception of why patients change compression therapies?
    - PROMPT: Do patients report a therapy being painful or uncomfortable? Did patients feel that it did not stay in place as well as your previous compression? More than once? Were patients experiencing difficulties with self-application (if relevant)? Did patients express a prefer for a different compression therapy?
    - PROMPT: Do you think patients have enough information on which to base decisions about changing compression therapies? Why is this? What could be done/what would work?

4. Education and Training

- What would help you with delivering optimal care for patients with venous leg ulcers? What prevents this from happening?
    - PROMPT: Is education and training sufficient? Are the available resources sufficient and appropriate?
    - PROMPT: How could the service be improved?
