# Supplementary material for: Compression therapies for the treatment of venous leg ulcers: study protocol for a process evaluation in a randomised controlled trial, VenUS 6
Source: Trials. 2023 Nov 14;24:727. doi: 10.1186/s13063-023-07681-7 (PMC10644437; doi:10.1186/s13063-023-07681-7)
Supplement: Supplementary file 3 — Additional file 3. Anonymous survey. [file 13063_2023_7681_MOESM3_ESM.docx]

1. Please record how easy you generally find applying the following compression therapies.

|  | Very Easy | Easy | Neutral | Difficult | Very difficult | N/A |
| --- | --- | --- | --- | --- | --- | --- |
| Four-layer bandage | ○ | ○ | ○ | ○ | ○ | ○ |
| Two-layer bandage | ○ | ○ | ○ | ○ | ○ | ○ |
| Compression wrap | ○ | ○ | ○ | ○ | ○ | ○ |
| Two-layer hosiery | ○ | ○ | ○ | ○ | ○ | ○ |

1. How easy do you think patient’s find applying wraps or two-layer hosiery themselves (or application by an informal carer)?

|  | Very Easy | Easy | Neutral | Difficult | Very difficult | N/A |
| --- | --- | --- | --- | --- | --- | --- |
| Compression wrap | ○ | ○ | ○ | ○ | ○ | ○ |
| Two-layer hosiery | ○ | ○ | ○ | ○ | ○ | ○ |

1. Please select how you normally access the following compression therapies (assume not made to measure). Multiple options possible.

|  | Prescription | Ordered from manufacturer | Held in stock | Other, namely |
| --- | --- | --- | --- | --- |
| Four-layer bandage | ○ | ○ | ○ | ○ |
| Two-layer bandage | ○ | ○ | ○ | ○ |
| Compression wrap | ○ | ○ | ○ | ○ |
| Two-layer hosiery | ○ | ○ | ○ | ○ |

1. How long does it normally take you to access the following treatments once you have decided to give to a patient?

|  | Immediately | Less than 3 days | 4 to 7 days | 8 to 14 days | More than 14 days |
| --- | --- | --- | --- | --- | --- |
| Four-layer bandage | ○ | ○ | ○ | ○ | ○ |
| Two-layer bandage | ○ | ○ | ○ | ○ | ○ |
| Compression wrap | ○ | ○ | ○ | ○ | ○ |
| Two-layer hosiery | ○ | ○ | ○ | ○ | ○ |

1. Do you have any other comments about compression therapy for treatment of venous leg ulcers?
